# Sjögren’s syndrome: a systemic autoimmune disease

**DOI:** 10.1007/s10238-021-00728-6

**Published:** 2021-06-07

**Authors:** Simone Negrini, Giacomo Emmi, Monica Greco, Matteo Borro, Federica Sardanelli, Giuseppe Murdaca, Francesco Indiveri, Francesco Puppo

**Affiliations:** 1 Department of Internal Medicine, Clinical Immunology and Translational Medicine Unit, University of Genoa and IRCCS Ospedale Policlinico San Martino, Viale Benedetto XV, 6, 16132 Genoa, Italy; 2 Department of Experimental and Clinical Medicine, University of Florence, 50134 Florence, Italy; 3 Department of Internal Medicine, University of Genoa, 16132 Genoa, Italy

**Keywords:** Sjögren’s syndrome, Xerostomia, Xerophthalmia, Ro/SSA antibody, La/SSB antibody

## Abstract

Sjögren’s syndrome is a chronic autoimmune disease characterized by ocular and oral dryness resulting from lacrimal and salivary gland dysfunction. Besides, a variety of systemic manifestations may occur, involving virtually any organ system. As a result, the disease is characterized by pleomorphic clinical manifestations whose characteristics and severity may vary greatly from one patient to another. Sjögren’s syndrome can be defined as primary or secondary, depending on whether it occurs alone or in association with other systemic autoimmune diseases, respectively. The pathogenesis of Sjögren’s syndrome is still elusive, nevertheless, different, not mutually exclusive, models involving genetic and environmental factors have been proposed to explain its development. Anyhow, the emergence of aberrant autoreactive B-lymphocytes, conducting to autoantibody production and immune complex formation, seems to be crucial in the development of the disease. The diagnosis of Sjögren’s syndrome is based on characteristic clinical signs and symptoms, as well as on specific tests including salivary gland histopathology and autoantibodies. Recently, new classification criteria and disease activity scores have been developed primarily for research purposes and they can also be useful tools in everyday clinical practice. Treatment of Sjögren’s syndrome ranges from local and symptomatic therapies aimed to control dryness to systemic medications, including disease-modifying agents and biological drugs. The objective of this review paper is to summarize the recent literature on Sjögren’s syndrome, starting from its pathogenesis to current therapeutic options.

## Introduction

Sjögren’s Syndrome (SS) is a systemic chronic autoimmune disorder of unknown etiology characterized by salivary and lacrimal glands immune-mediated damage, leading to dryness of the mouth (xerostomia) and eyes (xerophthalmia). Besides, dryness may affect other mucosal surfaces such as airways, digestive tract and vagina, resulting in the clinical picture of "sicca syndrome" or "sicca complex." As a systemic disease, SS can involve virtually any organ system, leading to extremely pleomorphic clinical manifestations. Concerning the impact of SS on quality of life, the disease negatively affects patient daily activity due to the high prevalence of fatigue, depression, anxiety and decreased physical performances. A recent study demonstrated that health-related quality of life scores of SS patients was comparable to those observed in other diseases like systemic lupus erythematosus (SLE) and rheumatoid arthritis (RA) that are generally considered more aggressive autoimmune disorders [1].

SS is defined as "primary" if it is not associated with other diseases or "secondary" if it occurs in association with an underlying autoimmune connective tissue disorder, such as RA, SLE or systemic sclerosis (SSc). SS owes its eponym to the Swedish ophthalmologist Henrik Samuel Conrad Sjögren (1899–1986) that firstly correlated the triad of keratoconjunctivitis sicca, xerostomia and polyarthritis in 1933 [2].

## Epidemiology

SS mainly affects women (average female to male ratio 9:1) of middle age [3]. SS is generally diagnosed in the fifth decade of life, with a mean age ranging from 51.6 (± 13.8) to 62 (± 13) years; however, first symptoms may occur years before diagnosis [4, 5]. According to a recent meta-analysis, the pooled incidence rate of SS was estimated to be 6.92 (95% CI, 4.98–8.86) per 100,000 people/year, while the prevalence rate was 60.82 (95% CI, 43.69–77.94) cases per 100,000 inhabitants [6]. Regarding the prevalence of SS in Europe, Cornec and Chiche have analyzed three robust epidemiological studies and estimated a combined prevalence of nearly 39/100,000 (0.04%) [7].

So far, only two studies addressed the influence of geolocation and ethnicity on the epidemiology of primary SS. A study conducted in the population of the Greater Paris area revealed that patients with non-European backgrounds had a twofold higher prevalence of SS as compared with those with a European background [8]. Moreover, the EULAR-SS Task Force analyzed data of 8310 patients from five continents. Authors observed that geolocation and ethnicity have a strong influence on the biologic and clinical phenotype of the disease. Concerning SS epidemiology, they reported that SS was diagnosed a mean of 7 years earlier in black/African-American compared with white patients, and the female-to-male ratio was highest in Asian patients (27:1) and lowest in black/African-American patients (7:1) [9].

A recent systematic review analyzed 10 studies involving 7888 patients with primary SS, addressing causes and predictive factors of mortality in SS [10]. Leading causes of death were infections, cardiovascular diseases and solid-organ and hematologic malignancies, but any statistically significant increase in the risk of mortality was found in SS patients compared with the general population. Nevertheless, it was not possible to exclude that patients with more severe disease may have an increased mortality risk. Risk factors potentially associated with increased mortality were older age at diagnosis, male gender, parotid enlargement, abnormal parotid scintigraphy, extra-glandular involvement, vasculitis, anti-SSB positivity, hypocomplementemia and cryoglobulinemia [10].

## Pathogenesis

Similarly to other autoimmune diseases, the etiology of SS is unknown [11]. To date, it is widely accepted that exposure to specific environmental factors in susceptible individuals may play a crucial role, thus, leading to the dysregulation of the immune system and disease occurrence. More specifically, a derangement of the innate immune barriers has a pivotal role in SS pathogenesis, especially in the early phases of the disease, through a mechanism involving the interferon (IFN) pathway [12, 13]. On the other hand, the adaptive immune system has a central role in SS development. Indeed, persistent B-cells activation and the proliferation of Th1 and Th17 cells contribute to the progression of the disease [14]. Finally, in recent years, researchers focused on epithelial cells functioning, which demonstrated to be relevant factors of this complex pathogenetic scenario [15]. Here, we will describe the main genetic, epigenetic and immunological alterations leading to the development of SS.

### Environmental factors

Different infectious agents, especially viruses, have been considered potential SS pathogenetic triggers [12]. Among them, Epstein–Barr virus (EBV) was found in lacrimal gland biopsies, as well as in saliva and salivary gland specimens [16, 17]. Interestingly, some authors found a correlation between EBV presence, disease severity and extra-glandular manifestations [16]. Indeed, EBV favors autoantigens release (i.e., the ribonucleoprotein complexes Ro/SSA and La/SSB) through epithelial cell apoptosis [18] and molecular mimicry mechanisms [19]. EBV is also able to activate the innate immune response, enhancing the IFN expression through the interaction with endosomal Toll-like receptors (TLRs) 3, 7 and 9 [18, 20, 21]. However, to date, no clear association with viral infections (EBV, hepatitis C virus, HTLV1 and Coxsackie A) has been found in SS [22]. Some studies suggested that, in genetically susceptible subjects, viruses may act as potential triggers, although they cannot be detected at the beginning of the clinical onset [23]. Finally, EBV has a well-established tropism for B cells, thus favoring chronic lymphoproliferation [24].

### Genetic and epigenetic factors

Genetic factors are supposed to play an important role in SS pathogenesis [11, 25, 26]. Familial association studies showed that about one-third of SS patients have a relative with another connective tissue disease [27]. In particular, twins have a higher risk of developing SS than subjects with sporadic SS [28]. More recently, associations between certain Human Leukocyte Antigen (HLA) alleles (e.g., HLA DRB1*03:01, DQA1*05:01, DQB1*02:01) and SS susceptibility have been demonstrated by genomic studies [29–31]. Among pathogenetic mechanisms involved in SS, interferon regulatory factor 5 (IRF5) polymorphisms have been studied too. Indeed, this protein is involved in the IFN I signaling and STAT4, thus regulating the type II IFN pathway [29]. Finally, other risks loci encoding molecules pivotal for the immune-pathogenetic mechanisms of the disease have been associated with SS. Among the others, the chemokine CXCR5, the B lymphoid kinase (BLK) and the Nuclear Factor (NF)-kB pathway are all involved in the control of B cell differentiation and proliferation and antibodies production [29, 32, 33]. The latter pathways have also been correlated with SS-associated lymphomagenesis [34].

It is widely accepted that epigenetic factors play an important role in autoimmune disorders. Although not completely defined, it is now quite evident that global DNA methylation affects epithelial cells of minor salivary glands [35–37]. Indirect evidence of epigenetic mechanisms involved in SS came from studies conducted on patients treated with the anti-CD20 antibody, rituximab. Among these subjects, B lymphocyte depletion was correlated with the restoration of DNA methylation in epithelial cells from labial salivary glands [36]. Taken together, epigenetic factors may regulate IFN pathways, micro-RNA signaling and genetic loci responsible for antigen presentation, thus contributing to SS occurrence [38].

### Epithelial cells

Nowadays, epithelial cells are considered major players in the pathogenesis of SS, so that the term autoimmune "epithelitis” is increasingly used to describe this condition [39]. Indeed, epithelial cells play a double role in SS pathogenesis, because they represent the target of the autoimmune process, but also the triggers of the immune activation [39]. The main processes regulated by epithelial cells are: a) the expression of ribonucleoprotein complexes (i.e., Ro/SSA and La/SSB), as a consequence of apoptotic mechanisms [40]; b) the interaction with T cells, via the expression on cellular surface of costimulatory proteins (i.e., CD86) [41, 42]; c) the production of cytokines (i.e., IL-21 and BAFF) to favor the development of T follicular helper, that in turn regulate B cells activity [43, 44]; d) the expression of chemokines (i.e., CXCL12), able to recruit leukocytes [45]. Interestingly, local inflammation and production of certain cytokines such as IFN-gamma and Tumor Necrosis Factor alpha may contribute to the secretory gland dysfunction observed in SS by disrupting tight junction structure of epithelial cells [46]. Alterations in the tight junction integrity lead to significant changes in salivary gland cell polarity and changes in cell organization may affect the localization and the functionality of the secretory machinery [47]. All these phenomena are potentially implicated in the reduction in the quality and quantity of saliva and may contribute to the development and maintenance of local inflammation observed in salivary glands of SS patients [46, 47].

### Innate immunity

#### Type I IFNs

An over-expression of IFN-inducible genes, also known as IFN-signature, was demonstrated among patients with SS, both in peripheral blood [48] and salivary glands [49, 50]. Intriguingly, an interaction between the IFN pathway and B lymphocyte activation has been proposed [51]. This cross-talk seems bi-directional, since B cells could induce the production of IFN, which in turn would be able to favor auto-antibodies production [48, 51]. Finally, a significant correlation between BAFF (both as serum level and mRNA expression) and type I IFN signature has been described in SS [52], thus representing a potential therapeutic target [53].

#### NK cells

In recent years, also Natural Killer (NK) cells have been implicated in SS pathogenesis. Indeed, it has been demonstrated that subsets of unconventional NK cells can produce inflammatory cytokines (i.e., IL-22) in salivary glands and could favor BAFF production [54]. Notably, it was recently suggested that SS patients with low numbers of NK cells in salivary glands had a better response to anti-BLyS treatment (belimumab), as compared to patients with higher levels of NK [55].

### Acquired immunity

#### B cells

B lymphocytes are considered major players in SS pathogenesis and its main complication (namely lymphoma) [56]. Indeed, several direct and indirect evidence of B cell activation in SS patients exists. In particular: (a) a significant increase in cytokines usually related to B cells (i.e., IL-6 and IL-10) has been demonstrated [57, 58]; (b) GC-like structures have been identified in the salivary glands of patients with SS and are able to promote chronic activation of autoreactive B lymphocytes [45]; (c) CXCL13 (a B lymphocyte chemoattractant) and its receptor CXCR5 are involved in the formation of GC-like follicles. These chemokines are all involved in the migration of B-lymphocytes into the B cell zone in salivary glands [45]. Also, in recent years the role of BAFF has strongly emerged. Indeed, its levels are significantly increased not only in the blood, where they correlate with the levels of anti-Ro/SSA and anti-La/SSB antibodies [59], but also in the salivary glands [60, 61]. Interestingly, BAFF is produced not only by immune cells but also by epithelial cells [60, 61]. Also, transgenic mice models have shown an overexpression of BAFF [62] and importantly, also an increased risk of lymphomagenesis [63].

As discussed above, EBV has been implicated in pathogenesis of SS. Interestingly, EBV can produce several proteins, including viral interleukin-10 (IL-10). IL-10 in turn can trigger the production of IL-6, interfere with the cell-mediated immune response and contribute to immortalization of B cells [64–66]. All these phenomena may play a potential role in SS immunopathogenesis including the development of SS-related lymphoma.

#### T cells

Besides B lymphocytes, also T cells significantly contribute to SS pathogenesis. As already mentioned, genetic studies proposed the role of Th1 cells in SS [31, 67]. However, based on other clues, Th1 cell polarization has been suggested. In particular, a significant increase in Th1-related cytokine (mainly IFN-gamma and IL-12) has been demonstrated both in mice models [68–71] and in humans [72]. More recently, Th17 cells have been studied too due to their emerging role in autoimmunity [73, 74]. Indeed, the presence of these cells has been directly demonstrated at the salivary gland level [75, 76], together with higher levels of IL-17 both in the serum and in the salivary tissue [67, 75]. Finally, although their role is still uncertain, also regulatory T cells (Tregs) could probably have a role in SS development [67, 77]. Tregs have been found within the salivary glands of patients with SS [78, 79], sometimes correlating with the focus score, thus suggesting their immune-regulatory activity at this level [79].

## Clinical manifestations

### Glandular manifestations

Ocular and/or mouth dryness are the symptoms most frequently complained by SS patients. Virtually all patients report at least one of them (98%), while 89% present both symptoms [4]. Symptoms of decreased salivary production include dysphagia, dysgeusia (altered taste sensation), pain and burning sensation. Patients usually complain of dry food swallowing impairment or the inability of speaking for long periods without drinking liquids. Physical examination of the mouth may reveal dry, erythematous oral mucosa, a lobulated or depapillated tongue, dental caries, periodontal disease, bacterial sialadenitis, *Candida* infection and angular cheilitis [5, 80]. Recurrent or chronic enlargement of the major salivary glands is also frequent, and it occurs in approximately one-third of the patients [81]. Glandular swelling usually involves parotid glands; however, submandibular or sublingual glands may be affected too. Glandular enlargement may start unilaterally, although it generally becomes bilateral [5, 81]. Therefore, in case of a persistent unilateral gland enlargement, clinicians should be prompt to research other causes [82]. Differential diagnosis of glandular swelling is recapped in Table 1. Although SS is rare among children, typical clinical presentation in pediatric SS is characterized by recurrent parotid gland enlargement, which often precedes sicca symptoms by years [83].

**Table 1:** Differential diagnosis of salivary gland enlargement

Cause	Notes
Sialadenosis (or sialosis)	Bilateral non-painful enlargement of the major salivary glands (typically the parotids) associated with systemic disorders (endocrine/metabolic, nutritional, drug-induced)
Sialolithiasis	Ductal obstruction causing pain and swelling of the affected salivary gland (typically unilateral, may complicate Sjögren's syndrome)
Infections	
Bacterial	Usually unilateral, painful swelling due to bacterial infection (e.g., acute suppurative parotitis), bacterial sialadenitis may complicate Sjögren's syndrome
Mycobacterial	Rare forms of extra-pulmonary tuberculosis
Viral	Acute viral parotitis—mumps (bilateral), HIV salivary gland disease, other viruses. HCV- infected patients may have histological signs of Sjögren-like sialadenitis
Wegener granulomatosis	May involve parotid glands (rare, bilateral or unilateral)
Sarcoidosis	May involve parotid or lacrimal glands. Heerfordt syndrome: uveitis, fever, parotid enlargement, facial palsy
Neoplastic (benign or malignant primary tumor)	Various histology; lymphoma (generally unilateral, may complicate Sjögren's syndrome)
IgG4-related disease	Lymphoplasmacytic infiltrate enriched in IgG4-positive plasma cells may affect lacrimal, parotid and submandibular gland (also termed Mikulicz syndrome)
Amyloidosis	May cause salivary gland enlargement
Masseteric hypertrophy	Asymptomatic enlargement of one or both masseter muscles (may mimic parotid enlargement)
Pneumoparotid	Passage of air through the parotid orifice and into the ducts in individual who increases intraoral pressure by forcefully blowing
Drugs	Rare (iodine-based contrast medium, radioactive iodine-131, anesthetics, others)

Lachrymal gland dysfunction causes qualitative and quantitative abnormalities of the tear film, thus leading to ocular surface chronic inflammation. Dry eye syndrome, also known as keratoconjunctivitis sicca, may cause a wide spectrum of signs and symptoms characterized by photosensitivity, erythema, itching or foreign body sensation [84]. Ocular symptoms are worsened by activities with a low blinking rate such as reading, using computer devices, driving or watching television. Moreover, eyes symptoms are worsened by windy, dusty, smoky  or dry environments [84]. Long-term complications of ocular involvement includes thickening of the corneal surface and corneal ulceration [84, 85]. Also, different medications (e.g., diuretics, beta-blockers, tricyclic antidepressants and antihistamines) may reduce salivary and lachrymal production thus aggravating keratoconjunctivitis sicca and oral symptoms in SS patients. It is worthy of note that symptoms of ocular and oral dryness are greatly more frequent than SS, especially among elderly people. Consequently, the differential diagnosis should include different conditions responsible for the reduction in tear and/or saliva secretion (Table 2).

**Table 2:** Differential diagnosis of conditions associated with mouth and/or eye dryness

Cause	Notes
Drugs (many)	Most common cause, especially among older patients. Mainly related to anti-cholinergic and/or sympathomimetic actions (e.g., antidepressants, benzodiazepines, antispasmodic agents, beta-blockers, antihistamines, diuretics, opioids, etc.). May worsen sicca symptoms in patients with Sjögren’s syndrome
Aging	Salivary flow and tears production decrease in an elderly patient (risk increased in subjects exposed to polytherapy)
Neuropathic	Reduced stimulation of exocrine glands (e.g., diabetes, Parkinson’s disease, Multiple Sclerosis)
Dehydration	Decreased saliva production (e.g., diabetes mellitus, diuretics, end-stage renal disease, impaired thirst perception, etc.)
Surgical removal of the salivary glands	Iatrogenic cause
Radiotherapy of the head and neck	Tear gland and/or salivary gland damage
Smoking	Long-term smoking significantly alters saliva flow rate and salivary pH
Alcohol abuse	Damage of mucosa and salivary glands
Dry/windy environment	Enhance tear and saliva evaporation
HCV sialadenitis	Can cause both salivary gland enlargement and reduced salivary production
HIV salivary gland disease	Related with lymphocytic infiltration, use of certain antiretroviral drugs
Stress, depression and anxiety	Influence sympathetic activity that controls saliva secretion
Chronic graft versus host disease	Related with lymphocytic infiltration, parenchymal damage and fibrosis
Amyloidosis	Amyloid infiltration and destruction of salivary glands
Sarcoidosis	Granulomatous infiltration may lead to xerostomia

Symptoms related to other exocrine glands dysfunction are relatively common [5]. Respiratory tract dryness may cause persistent hoarseness and chronic non-productive cough [86]. Skin involvement is characterized by cutaneous xerosis, whereas reduced vaginal secretion leads to dyspareunia and local discomfort [5, 87]. Besides, diminished secretion of the exocrine glands of the digestive tract may involve pancreas and stomach causing pancreatic dysfunction and hypochlorhydria, respectively [88].

### General constitutional symptoms

Fatigue is reported by about 70–80% of SS patients, and it often has a negative impact on their quality of life. Other non-specific general symptoms are sleep disorders, anxiety, depression and chronic widespread pain [89]. Some SS patients, typically middle-aged women, present with a clinical triad characterized by dryness, pain and fatigue. Interestingly, this subgroup of patients shows less systemic manifestations and a lower prevalence of autoantibodies [89]. In this case, the differential diagnosis between SS and other potential disorders, such as menopause, hypothyroidism, diabetes, malignancy, depression or fibromyalgia, may be particularly challenging.

### Musculoskeletal involvement

Musculoskeletal involvement, comprehending myalgia, arthralgia and morning stiffness are present in the majority of SS patients [90]. The frequency of joint pain without inflammatory signs (arthralgia) is reported up to 75% of patients [4], whereas overt inflammatory joint disease (arthritis) is less frequent and can be observed in approximately 10% of SS patients [4, 81]. Arthritis is predominantly symmetrical, generally mono- or oligoarticular with the involvement of proximal interphalangeal, metacarpophalangeal joints and wrists [91]. The large majority of SS-related arthritis is non-erosive, while synovitis and bone erosions are characteristic features of secondary SS associated with RA [90, 91]. In SS patients, true myositis is rare, while myalgias and/or muscle weakness are relatively frequent and can be secondary to fibromyalgia or hypokalemia, respectively [92, 93].

### Dermatological involvement

Besides xerosis (skin dryness), which is the commonest cutaneous manifestation of SS, other manifestations of skin involvement are relatively frequent [91]. Annular erythema is a non-scarring, not–atrophy-producing, dermatosis characterized by annular polycyclic lesions typically occurring in photo-exposed areas and it is characterized by a wide elevated border and a central pallor area [94]. In analogy with subacute cutaneous lupus erythematosus, SS-related annular erythema is strongly associated with the positivity of anti-Ro/SS-A and/or anti-La/SS-B autoantibodies [12, 91]. Cutaneous vasculitic lesions are mostly represented by palpable purpura, most commonly distributed over the lower limbs, but other clinical entities such as cutaneous ulcers, urticarial vasculitis or skin nodules have been reported [91]. Skin biopsy is not mandatory for the diagnosis, but when it is performed, it shows leukocytoclastic vasculitis in the majority of the cases. Patients with vasculitis present a high prevalence of anti-Ro/SSA and/or anti-La/SSB antibodies and about one-third of them has a positive cryoglobulin test [91]. Raynaud phenomenon is reported in 10–20% of SS patients, and in the majority of the cases, it precedes the onset of sicca symptoms [5, 95]. Typically, its clinical course is milder than in SSc [96].

### Respiratory tract involvement

Respiratory tract involvement is often described among SS patients. The prevalence of pulmonary involvement in patients with SS is estimated between 9 and 24%, but subclinical abnormalities of pulmonary function tests (PFTs), bronchoalveolar lavage (BAL) and computed tomography (CT) can be identified in up to 75% of patients [86]. Upper airways dryness can promote nasal crusting, epistaxis and rhinosinusitis. Thick mucus at vocal cords may cause chronic hoarseness and approximately 50% of SS patients complain chronic non-productive cough resulting from tracheal dryness (xerotrachea) [86]. Moreover, airways dryness predispose SS patients to atelectasis, bronchiectasis and recurrent episodes of respiratory tract infections [86].

Regarding distal airways involvement, the most typical manifestation is bronchiolitis (mainly follicular bronchiolitis) [97]. Respiratory complications of SS also include a variety of interstitial lung diseases (ILDs) such as non-specific interstitial pneumonia (the most common histopathological pattern), organizing pneumonia, lymphocytic interstitial pneumonia and cryptogenic organizing pneumonia [86, 97]. From a clinical point of view, ILDs are characterized by cough and dyspnea. Typical radiologic findings on high-resolution CT together with a restrictive ventilatory defect and a reduced diffusing capacity for carbon monoxide (DLCO) at PFTs, strongly suggest the presence on an ILD [86, 97, 98]. Other respiratory complications described in SS patients include amyloidosis, BALT (bronchus-associated lymphoid tissue) lymphomas, thromboembolic disease, pulmonary arterial hypertension and pleural disease [86, 99].

Considering the frequency and potential severity of pulmonary disease in SS, experts recommend a systematic screening of all patients [97, 99]. In this context, clinical practice guidelines have been recently developed by the Sjögren's Foundation in order to help clinicians to early identify and manage SS-related pulmonary manifestations [100]. In general, all asymptomatic patients should undergo a baseline chest radiograph and serial clinical and PFTs monitoring. In patients with respiratory symptoms, complete PFTs and HRCT should be done to evaluate for pulmonary involvement, and, in certain cases, further tests (e.g., echocardiogram, CT pulmonary angiogram or bronchoscopy) may be required to clarify the underlying cause of symptoms.

### Cardiac involvement

Clinically overt cardiac disease is rarely observed in SS. Nevertheless, different studies reported an increased prevalence of the valvular disease, left ventricular abnormalities, pulmonary hypertension and pericardial effusion in SS patients as compared to a normal population [98, 101, 102].

### Nervous system involvement

Nervous system involvement can be observed in up to 20% of SS patients, sometimes anticipating the sicca symptoms. SS may affect both central (CNS) and peripheral nervous systems (PNS) [5, 103, 104].

The latter is usually the most involved, and sensory ataxic neuronopathy and painful small fibers neuropathy are the two most typical forms of SS-associated neuropathies. Therefore, SS should be considered as a differential diagnosis among patients presenting with these neurological entities [103]. Sensory ataxic neuronopathy is related to a dorsal root ganglionitis characterized by T-cell infiltration and loss of neuronal cells of the dorsal root ganglia. From a clinical point of view, patients display loss of kinesthesia and proprioception leading to sensory ataxia, difficulty with fine motor movements, unsteady gait and reduced or absent reflexes [103, 105]. The painful sensory neuropathy is a small fibers neuropathy characterized by symmetric distal dysesthesias with paresthesias, allodynia and hyperalgesia. Typically, painful sensory neuropathies do not have electrophysiologic abnormalities; therefore, if suggestive symptoms are present, the diagnosis should be confirmed with a skin biopsy demonstrating a reduced density of epidermal nerve fibers [103, 105]. Additional PNS disorders observed in SS patients include sensorimotor polyneuropathy, autonomic neuropathy, mononeuritis multiplex and cranial neuropathies [104].

In SS patients, CNS disorders are less common than peripheral neuropathies. Moreover, they include a variety of manifestations that can be difficult to differentiate from other neurological diseases. Virtually all CNS structures may be affected, including the spinal cord, brainstem, optic nerves, cerebellum and cerebral hemispheres [104]. Recently, an association between SS and neuromyelitis optica (NMO) has been reported. NMO is a CNS demyelinating relapsing–remitting disease characterized by inflammation and demyelination of the optic nerve (optic neuritis) and the spinal cord (myelitis) associated with the presence of autoantibody against aquaporin-4 [103, 106]. Clinical manifestations of NMO are acute episodes of painless visual loss due to the optic neuritis, also culminating in a complete and bilateral loss of view and other symptoms suggestive of transverse myelitis such as limb weakness, sensory loss and bladder dysfunction [107]. Diagnosis is confirmed with MRI imaging, analysis of cerebrospinal fluid and positivity of specific autoantibodies. MRI specific lesions suggestive of NMO may also occur outside the optical nerve and spinal cord, especially in the brain [103, 107]. Among CNS disturbances, cognitive dysfunction and sleep disorders are commonly observed among SS patients. These conditions contribute to worsen overall patient’s quality of life [108, 109].

### Renal involvement

The prevalence of the renal disease in SS has been reported to be approximately 5%, but it is probably underestimated [91, 110, 111]. Chronic tubulointerstitial nephritis is the predominant form of SS-associated renal involvement, which clinically translates mostly into distal (type I) renal tubular acidosis (RTA). Distal RTA is characterized by a cortical collecting duct dysfunction that involves the acid-secreting alpha-intercalated cells leading to an impaired H+ elimination [110, 111]. The defect may be complete, with systemic metabolic acidosis, or incomplete, characterized by the inability to acidify urine following an oral acid loading challenge. In symptomatic patients, distal RTA presents with weakness/paralysis due to hypokalemia and less frequently with renal calculi and osteomalacia [110, 111]. Mildly elevated serum creatinine and low-grade proteinuria may be present [91, 110, 111]. Other less common forms of SS-related tubulointerstitial nephritis are represented by dysfunctions involving cortical collecting duct (principal cells), proximal tubular, loop of Henle and distal convoluted tubule [110, 112].

In contrast to tubulointerstitial involvement, the glomerular disease is less common and rarely constitutes the initial manifestation of the disease [5]. In the majority of cases, SS-related glomerulonephritis is a membranoproliferative glomerulonephritis caused by a non-infective cryoglobulinemic vasculitis characterized by deposition of immune complexes [110, 111]. The SS-related glomerular disease generally emerges from abnormal routine analyses (proteinuria, elevated serum creatinine) rather than from clinical symptoms (edema/nephrotic syndrome) [91, 110–112].

### Gastrointestinal involvement

Studies suggest that up to 80 percent of SS patients may experience some degree of dysphagia, with negative consequences on their quality of life. Dysphagia may derive from the combination of xerostomia and/or esophageal dysmotility [113]. Gastric and bowel motility disorders can be related to the presence of autonomic neuropathy and stomach involvement; however, they may be also due to the reduction in acid production, anti-parietal antibodies and gastritis [113]. Subclinical pancreatic dysfunction, characterized by exocrine pancreatic impairment with reduced production of amylase and lipase, can be observed in SS patients [88, 113]. Abnormal liver function tests can be detected in up to 50% of patients, but only a minority of them have a frank liver disease such as primary biliary cholangitis, autoimmune hepatitis or non-alcoholic fatty liver disease [88, 113]*.*

### Associated organ-specific autoimmune disorders

SS can be defined as secondary when it is associated with other systemic autoimmune diseases. Nonetheless, different organ-specific autoimmune disorders can be observed in SS patients. Celiac disease can be diagnosed in up to 15% of patients with SS [113, 114]. Autoimmune hepatitis and primary biliary cholangitis have been reported in up to 10% of patients with SS [113, 115]. Hypothyroidism and Grave’s disease are reported in 7–14% and 1.8–3% of patients with SS, respectively [115]. Antiphospholipid antibodies (aPL) can be found in up to 13% of SS patients, whereas clinical overt antiphospholipid syndrome develops in only 10% of aPL-positive SS patients [116, 117].

### Lymphoma and other hematologic disorders

Lymphoma is one of the most severe complications of SS, indeed approximately 5% of SS patients develop lymphoma [4]. A recent meta-analysis estimated that SS patients have a relative risk of developing lymphoma of 13.76 (95%, CI 8.53–18.99) [118]. SS-associated lymphomas are typically low-grade B cell non-Hodgkin lymphomas (NHLs), in particular MALT lymphoma, which accounts for 60% of cases, nodal marginal zone lymphoma and diffuse large B-cell lymphoma. Lymphomas often develop in organs where the disease is active, and salivary glands are the most frequent sites [119]. NHLs complicating SS tend to have an indolent course without B symptoms (fever, night sweats and weight loss) and only 10% of them might transform into a less differentiated (more aggressive) variety [119]. Among SS patients, different risk factors have been associated with increased susceptibility to develop lymphoma. The main clinical predictive factors are persistent swelling of salivary glands, lymphadenopathies and palpable purpura [119]. Additionally, a moderate to high disease activity has been independently associated with subsequent lymphoma occurrence [120]. The main biological predictors of SS-related lymphoma are cryoglobulinemia, lymphopenia, hypocomplementemia and the presence of a serum and/or urinary monoclonal component [119]. Moreover, the detection of germinal center (GC)-like structures in salivary glands biopsies is highly predictive of SS-associated lymphoma [121]. All these predictors are easy to evaluate in daily routine; consequently, they should be included in the management of every SS patient. Nevertheless, to date, there are no specific guidelines on the frequency of their monitoring [119, 122]. Treatment of SS-associated lymphoma is related to stage at diagnosis, involved site(s) and histopathologic features and can vary from active monitoring strategy to multiple combined chemotherapy [122].

A reduction in the number of blood cells can be observed in one-third of patients with SS [5]. SS-related cytopenia include normocytic anemia, leukopenia and thrombocytopenia and are more commonly described in patients with positive immunologic markers. The overwhelming majority of SS-associated cytopenia are asymptomatic [123].

### Pregnancy

SS does not directly impair fertility; however, pregnancy complications among SS patients are described. Indeed, SS affected women present an increased rate of miscarriage and preterm deliveries. Moreover, they give birth to babies with lower birth weight and have complications during deliveries more frequently than unaffected women [124]. In particular, SSA/Ro and/or SSB/La positive women have an increased risk of delivering an infant affected by Neonatal Lupus Syndrome (NLS). NLS is an acquired autoimmune disease caused by transplacental passage of maternal anti-SSA/Ro and/or anti-SSB/La autoantibodies to the fetus [125]. Clinical manifestations of NLS include a transitory skin rash, cytopenia, hepatic involvement or congenital heart block (CHB). CHB occurs in < 2% of pregnancies, and it is the most severe fetal complication as it is associated with an overall mortality rate of 22.9% and needs for pacemaker implantation in 79.1% of cases [126]. Interestingly, CHB may complicate pregnancies of asymptomatic autoantibodies-positive women, thus preceding the onset of the overt autoimmune disease [127]. CHB can be detected by ultrasound scanning from about 16 weeks’ gestation; consequently, autoantibodies-positive mothers should be strictly monitored by serial echocardiograms during pregnancy [127, 128].

## Diagnosis

Since 1965, several classification criteria sets have been proposed. More recently, two sets of criteria have been published. The first was the 2002 revised American–European Consensus Group (AECG) Classification Criteria. These criteria have been largely employed for research and clinical purposes [129]. The second set of criteria was published by the Sjögren’s International Collaborative Clinical Alliance (SICCA) and endorsed by the American College of Rheumatology (ACR) in 2012 [130]. Despite the differences between the ACR and AECG classification criteria, in 2014 Rasmussen and colleagues compared the performances of the two sets of criteria and found a high level of concordance, but also no clear evidence for the increased value of the new ACR criteria over the old AECG criteria [131].

The lack of an international consensus on SS classification criteria, prompted the international scientific community to develop a novel set of criteria for SS. As consequence, in 2016 the ACR and the European League Against Rheumatism (EULAR) developed and validated a novel set of classification criteria for SS combining items from both the AECG and ACR criteria [132]. It is worth underlining that these criteria have been elaborated for improving patient recruitment in clinical trials, consequently, they are focused on primary SS because patients with secondary SS are generally excluded from experimental protocols [132]. The three above-cited classification criteria are schematically summarized in Table 3. Recently, the performance of the new ACR/EULAR criteria has been compared to alternative sets of SS classification criteria, demonstrating higher sensitivity and lower specificity in identifying SS patients [133, 134]. Probably the introduction of novel biomarkers and/or the inclusion of salivary gland ultrasonography will further improve the performances of the currently available criteria. The 2016 ACR /EULAR classification criteria apply to any patients with at least one symptom of ocular or oral dryness (based on AECG questions) or suspicion of SS based on the positivity of at least one of the domains of the ESSDAI questionnaire (see below) [132]. According to these criteria, a subjects may be classified as having primary SS if he/she has a total score of ≥ 4, obtained from the sum of five objective items: anti-SSA/Ro antibody positivity and/or focal lymphocytic sialadenitis with a focus score of ≥ 1 foci/4 mm^2^, each scoring 3; an abnormal Ocular Staining Score (OSS) of ≥ 5 (or van Bijsterveld score of ≥ 4), a Schirmer’s test result of ≤ 5 mm/5 min and an unstimulated salivary flow rate of ≤ 0.1 mL/min, each scoring 1 [132].

**Table 3:** Comparison of 2002, 2012 and 2016 classification criteria for Sjögren's syndrome [125, 126, 128]

2002 American European Consensus Group (AECG)	2012 American College of Rheumatology (ACR)	2016 ACR/Eular Classification Criteria
Item I. Ocular symptoms (daily, persistent, troublesome dry eyes for more than 3 months and/or recurrent sensation of sand or gravel and/or use tear substitutes more than 3 times a day)	1. Anti-Ro/SSA and/or anti-La/SSB OR positive rheumatoid factor + ANA ≥ 1:320	1. Labial salivary gland biopsy (focal lymphocytic sialoadenitis a focus score ≥ 1); score 3
Item II. Oral symptoms (daily feeling of dry mouth more than 3 months and/or recurrently or persistently swollen salivary glands as an adult and/or need of liquids to swallow solid food)	2. Labial salivary gland biopsy (focal lymphocytic sialoadenitis with a focus score ≥ 1)	2. Anti-Ro/SSA positivity; score 3
Item III. Ocular signs (Schirmer's test no anesthesia and/or rose Bengal or other dye score (> 4 according to van Bijsterveld’s scoring system)	3. Keratoconjunctivitis sicca (ocular staining score ≥ 3 according to Whitcher’s protocol; exclude: use of glaucoma eye drops, corneal or cosmetic eyelid surgery in the last 5 years)	3. Ocular Staining Score ≥ 5 according to Whitcher’s protocol (or van Bijsterveld’s score ≥ 4) in at least 1 eye; score 1
Item IV. Histopathology (focal lymphocytic sialoadenitis in minor salivary glands with a focus score ≥ 1)	Classification (only for primary SS): individuals with signs/symptoms suggestive of SS + at least 2 of the 3 objective features	4. Schirmer’s test < 5 mm/5 min in at least 1 eye; score 1
Item V. Salivary gland (unstimulated salivary flow ≤ 1.5 mL/15 min and/or parotid sialography with diffuse sialectasias without obstruction and/or salivary scintigraphy specific abnormalities	Exclusion: history of head and neck radiation treatment, HCV, AIDS, sarcoidosis, amyloidosis, GVHD, IgG4-related disease	5. Unstimulated whole saliva flow rate (< 0.1 ml/minute, as described by Navazesh and Kumar); score 1
Item VI. Autoantibodies (anti-Ro/SSA and/or La/SSB)		Classification: a score ≥ 4 from the five criteria items in any patient with at least one symptom of ocular (see item I of the AECG 2002 criteria) or oral dryness (see item II of the AECG 2002 criteria, excepting “salivary glands enlargement”) or in whom there is suspicion of SS from the European League Against Rheumatism SS Disease Activity Index (ESSDAI) questionnaire (at least one domain with a positive item)
Classification: primary SS (any 4 of the 6 items, with positive item IV or VI OR any 3 of the 4 objective criteria (III to VI); secondary SS (potentially associated disease + presence of item I OR item II + any 2 among items III to V)		Exclusion: history of head and neck radiation treatment, HCV, AIDS, sarcoidosis, amyloidosis, GVHD, IgG4-related disease
Exclusion: history of head and neck radiation treatment, HCV, AIDS, preexisting lymphoma, sarcoidosis, GVHD, anticholinergic drugs		

### Autoantibodies

Antinuclear antibodies (ANAs), rheumatoid factor (RF), and Ro/SSA and La/SSB autoantibodies are typical serological findings in SS. ANA are present in the sera of up to 85% of patients, and also RF is commonly positive in SS; for this reason, they had been included as SS criteria in different previous classification sets, nevertheless they were considered too unspecific and consequently they have been excluded from the 2016 ACR/EULAR criteria [135]. Also, it should be noted that some patients may display anti-Ro/SSA and/or anti-La/SSB antibodies despite a negative immunofluorescence ANA test. Anti-Ro/SSA and anti-La/SSB antibodies are considered the key immunological markers of SS and can be found in 33–74% and 23–52% of SS patients, respectively [115]. Different studies have shown that the positivity for these autoantibodies is associated with early disease onset, longer disease duration and more severe glandular involvement. Besides, their presence correlates with the occurrence of extra-glandular systemic manifestations and with the risk of neonatal lupus and CHB in the fetus [115]. The new 2016 ACR/EULAR criteria have excluded isolated anti-SSB/La positivity among the items since anti-SSA/Ro antibodies are usually detected either solely or concomitantly with anti-SSB/La, whereas anti-SSB/La positive-SSA/Ro negative patients are rare [132, 135]. Lastly, it should be noted that an isolated finding of anti-SSA/Ro and/or SSB/La is not sufficient to make a diagnosis of SS, as these autoantibodies can be found in other connective tissue diseases and even in healthy subjects [136].

### Salivary gland biopsy

Labial salivary gland biopsy is the key test to confirm SS diagnosis and it may be decisive in patients with sicca symptoms without anti-SSA/Ro antibodies. Also, it provides prognostic information since a high degree of lymphoid infiltration does increase the risk of lymphoma development [137]. Minor salivary gland biopsy is a simple procedure that can be performed under local anesthesia and with a low rate of side effects. Examination of the tissue sections should be performed by a pathologist with experience in focal lymphocytic sialadenitis and focus score count (number of foci with 50 or more mononuclear cells per 4 mm^2^), following a standardized protocol such as those described by Daniels et al. [138]. Furthermore, an optimal pathology report should include the evaluation of the presence of germinal center-like structures and IgG4 staining to exclude an IgG4-related disease [139]. Parotid gland biopsy is generally not necessary for the diagnosis of SS, but it may be indicated to investigate atypical parotid enlargement suggestive of neoplastic pathology.

### Schirmer's test

Schirmer's test is a simple procedure used to assess the amount of lachrymal production. The test is performed by placing a strip of sterile paper in the lateral third of the lower eyelid of each eye and then measuring the length of the moistened portion of the strip. A Schirmer’s test result ≤ 5 mm/5 min in at least one eye is considered positive [132].

### Ocular staining

Evaluation of the ocular surface is performed by an experienced ophthalmologist through the instillation of topical dyes to determine the integrity of the epithelium layers of the cornea and conjunctiva. Fluorescein is used to determine the integrity of the corneal epithelium, while lissamine green (or rose Bengal) is used for evaluating the integrity of the conjunctiva. According to the 2016 ACR/EULAR criteria, an Ocular Staining Score ≥ 5 [140](or a van Bijsterveld score ≥ 4 [141]) in at least one eye is considered significative [132].

### Sialometry

Unstimulated whole saliva production is evaluated by collecting the patient's saliva in a calibrated tube for 15 min following the protocol for saliva collection redacted by Navazesh and Kumar [142]. An unstimulated whole saliva flow rate ≤ 0.1 mL/min is considered pathologic [132].

Similarly to other complex autoimmune diseases, the diagnosis of SS is based upon the judgment of an experienced clinician after the exclusion of alternative diagnoses. A complete medical history and a full physical examination are necessary in order to identify symptoms and signs suggestive of exocrine and systemic manifestations of SS. Then, clinical findings will be integrated with laboratory and/or specific tests in order to confirm the clinical suspicion. SS classification criteria may help clinicians to formulate the diagnosis of SS and facilitate differential diagnosis with other autoimmune or non-autoimmune disorders mimicking SS.

## Disease severity and activity measures

The necessity of specific and validated tools to measure outcomes in clinical trials on SS has prompted the EULAR Sjögren’s task force to develop two disease activity indices: the EULAR Sjögren’s Syndrome Patient Reported Index (ESSPRI), a patient-administered questionnaire assessing patient's subjective symptoms [143], and the EULAR SS Disease Activity Index (ESSDAI), a clinical index that measures systemic disease activity [144]. ESSPRI is a very simple questionnaire in which patients are asked to assess the severity of dryness, fatigue and pain experienced during the preceding 2 weeks. For this purpose, ESSPRI questionnaire includes three different 10-point Likert scales and patients answers may range from 0 (absence of symptom) to 10 (maximum imaginable intensity) [143]. The ESSDAI includes 12 domains (constitutional, lymphadenopathy, glandular, articular, cutaneous, pulmonary, renal, muscular, peripheral nervous system, central nervous system, hematological and biological), divided into 3–4 levels of activity [144]. Recently, the EULAR-SS Task Force has published a complementary glossary of systemic definitions included in the ESSDAI in order to ensure a more accurate and reproducible rating of each domain [145]. Both ESSPRI and ESSDAI scores have been validated and they demonstrated to have a high content validity, to be highly reproducible and to be able to detect change in disease activity [146]. The latest outcome measure elaborated by the EULAR-SS Task Force is the clinical ESSDAI (ClinESSDAI). ClinESSDAI does not evaluate the biological domain of the ESSDAI and weighs the other domains differently [147]. According to the authors, ClinESSDAI could be useful in biological/clinical studies to avoid data collinearity, in clinical trials to detect change independent of the biological effect of the drug and in clinical practice to assess disease activity when immunological tests have not been carried out [147].

## Treatment

Since a curative treatment of SS is currently unavailable, the goal is to alleviate symptoms of the exocrinopathy, as well as controlling the extra-glandular manifestations of the disease. Management of SS patients requires an integrated multidisciplinary approach, including different health care figures, such as family physicians, clinical immunologists or rheumatologists, ophthalmologists, otorhinolaryngologists and/or dentists. Depending on the systemic involvement, consultation with other specialists (e.g., pulmonologist, neurologist, gynecologist, etc.) may be indicated. Recently, different recommendations for the treatment of Sjögren's disease have been released. The Sjögren's Syndrome Foundation developed the first US clinical practice guidelines for the management of the oral, ocular and rheumatologic/systemic manifestations [148–151]. The British Society for Rheumatology has published a guideline for the management of adults with primary SS including several indications on the treatment of glandular and systemic manifestations of SS [128]. In 2019 the EULAR study group released updated recommendations for the management of SS with topical and systemic therapies [152]. The authors of these recommendations do not provide a specific therapeutic goal, apart from symptoms relief. Dryness should be managed with topical therapy as first step approach, sparing systemic therapies for active disease, whereas systemic manifestations should be treated with a targeted-organ approach with subsequent therapeutic steps [152]. Steroids need to be administered at the minimum dose and length of time in order to achieve disease control. Immunosuppressive drugs can be used as steroids sparing agents. B lymphocyte targeted strategies (e.g., rituximab, epratuzumab and belimumab) are reserved to patients with severe and refractory systemic disease [152]. Here, we want to summarize the main indications reported in the currently available literature.

### Sicca symptoms

Treatment of sicca symptoms is crucial to improve the quality of life of SS patients. On the whole, the management of SS-related dryness is based on pharmacological and non-pharmacological treatments aiming to preserve, substitute and stimulate glandular secretions while reducing local inflammation.

Ocular dryness is the symptom with a greater impact on the quality of life among SS patients [149]. As a general principle, the management of dry eye should be tailored to the severity of the condition and the patient's response to each adopted treatment. Patients with mild dry eye can be managed with patient education through simple advice on lifestyle habits [148, 149, 152]. Avoiding dry or windy environments and wearing protective eyeglasses or goggles can be helpful. Limiting prolonged activities associated with reduced blink rate, such as reading, driving or using a computer, may improve eyes discomfort. Moreover, limiting, or avoiding when possible, medications that reduce tear production (Table 2) can prevent the exacerbation of the dry eye symptoms. Lastly, maintaining good ocular hygiene and lid massage with warm compresses may reduce blepharitis and meibomian gland dysfunction [148, 149, 152]. In selected patients with blepharitis, topical antibiotics or low dose oral tetracyclines may be indicated to decrease bacterial colonization of the lid margins [128, 149, 152]. First-line pharmacological treatment for SS-related dry eye is based on artificial tear drops and lubricating ointments [149, 152]. Teardrops are administered on as-needed basis, but research suggests that regular dosing produces better results [84]. Ophthalmic ointments are the thickest among lubricants used to protect the ocular surface, and they are typically used before bedtime since they can blur vision [128, 149, 152]. Keratoconjunctivitis sicca is characterized by an inflammatory response on the ocular surface, so it is not surprising that different studies have demonstrated the clinical value of the use of topical steroids in managing SS patients with the moderate or severe ocular surface disease [84, 128, 149]. Nevertheless, due to the possible complications associated with steroid applications, such as cataract, glaucoma, their use should be limited to the short-term course in those patients who have failed to respond adequately to other therapies [128, 149, 152]. Topical cyclosporine, alone or in association with topical steroids, has demonstrated to provide a significant improvement of ocular signs and symptoms associated with chronic inflammation [84, 128, 149, 152]. Oral pilocarpine and cevimeline are muscarinic receptor agonist acting as lacrimal and salivary gland secretagogues, that have demonstrated to be effective in reducing dry eye symptoms, and probably effective in improving objective signs of dry eye [128, 149]. Patients' refractory to medical therapy and maximal lubrication may benefit from punctal occlusion. Punctal occlusion consists of the blockage of the tear drainage system at the level of the *puncta lacrimalia* through the insertion of plugs (temporary) or cauterization (permanent). This procedure aims to prevent the drainage of tears (or instilled tears substitutes) from the ocular surface [128, 149, 152]. Patients with severe dry eye and corneal ulceration, not responding to standard therapy, should be referred to a specialist center for consideration of scleral contact lenses, closure of the interpalpebral fissure or corneal grafting. If available, severe cases of ocular surface damage have been successfully treated with topical autologous serum [128, 149].

Dry mouth is a very common and annoying symptom in SS patients. As a preventive measure, all patients should be instructed to avoid factors that aggravate dry mouth symptoms, such as xerogenic medications and caffeine, tobacco smoking and alcoholic drinks [153, 154]. Moreover, a concomitant disease that can lead to mouth breathing and further drying of the oral cavity (e.g., sinusitis or rhinitis) should be treated [149]. Local measures used to reduce oral dryness symptoms include drinking, sipping or gargling frequently with water during the day, use mechanical/gustatory stimulating agents (e.g., sugar-free candies or chewing gums) to increase salivary flow, humidify the environment, and, if necessary, use a saliva substitute to maintain oral lubrication [128, 152, 154]. Muscarinic agonists, such as pilocarpine or cevimeline, may be beneficial in patients with significant sicca symptoms with residual salivary gland function [128, 150, 154]. Cholinergic agonists can cause some side effects like nausea, flushing, sweating and should be administered with caution in patients with cardiac disease or asthma because of the risks of bradycardia and bronchospasm [128]. Patients with SS have a well-known increased risk of developing caries and periodontal disease [148, 150]. Accordingly, patients should be encouraged to have regular dental examinations, reinforce oral hygiene, reduce acidic or sugary foods (and drinks) intake, take the adequate measures to increase salivary flow (see above) and use fluoride-containing toothpaste, mouth wash and gel [128, 150, 154]. Fungal oral infection, frequently observed in SS patients, can be managed by increasing oral hygiene and using topical antimycotic agents [128]. Antimicrobial agents, such as chlorhexidine (gel, varnish or mouth wash), may be indicated in patients with high caries rate and/or gum disease [150, 154].

The management of other sicca symptoms such as xeroderma or dry nose includes the use of skin emollients and nasal lubricants as required. Topical lubricants and estrogens are recommended for the local symptomatic treatment of vaginal dryness. Systemic secretagogues may be beneficial also for systemic dryness [128].

### Extra-glandular disease

To date, therapeutic recommendations for the treatment of systemic manifestations of SS are mainly empirical and are generally based on management strategies used for closely related systemic autoimmune diseases such as SLE and RA.

Fatigue represents one of the main symptoms negatively affecting the quality of life in SS patients and the efficacy of current medical treatments to control this symptom is still unsatisfactory. To date, regular physical activity is probably the most useful approach to ameliorate fatigue in SS [128, 151].

Hydroxychloroquine (HCQ) is generally recommended as a first-line treatment for skin and/or musculoskeletal pain [128, 152]. Conversely, a recent meta-analysis showed that there is no significant difference between HCQ and placebo in the treatment of ocular or mouth dryness in SS patients [155, 156].

Methotrexate (MTX), alone or in association with HCQ, is recommended for musculoskeletal involvement non-responsive to HCQ alone, in particular, those patients with significant inflammatory arthritis [128]. If the association of HCQ and MTX is not effective in the treatment of inflammatory musculoskeletal manifestations, alternative options, including corticosteroids, leflunomide, sulfasalazine, azathioprine, or cyclosporine, may be considered [151, 152].

Corticosteroids are generally not recommended for uncomplicated SS; however, they may be indicated, alone or in conjunction with other immunosuppressive agents, for important organ manifestations, such as lung disease, cytopenia and neurological involvement [128, 152]. Low-dose oral corticosteroids may be considered in patients with persistent constitutional or inflammatory musculoskeletal symptoms who have failed to respond to HCQ or other immunosuppressive drugs [128, 151, 152].

Azathioprine, or mycophenolate, may be indicated in patients with systemic complications, such as lung disease, myelopathy and cytopenia. Cyclophosphamide, commonly in association with corticosteroids, may be considered in patients with life-threatening or organ-threatening systemic complications such as the CNS, renal or lung disease [128, 151, 152].

B-cell hyperactivity plays a well-known role in the immunopathogenesis of SS; therefore, B-cell targeting treatments represent an intriguing therapeutic approach. There is accumulating evidence from published case studies and case series supporting the use of rituximab, an anti-CD20 B lymphocyte-depleting agent, in patients with active, systemic SS [152, 157]. To date, rituximab may be considered in patients with sicca manifestations who failed to respond to maximal conventional therapies [151]. Also, rituximab may be effective in the treatment of different SS-related systemic manifestations, including vasculitis, severe parotid swelling, inflammatory arthritis, lung disease, nephropathy, peripheral neuropathy and lymphoma [128, 151, 152, 158]. Belimumab, a human monoclonal antibody that inhibits B cell-activating factor (BAFF), has shown promising preliminary results supporting its use in SS, but further investigations are still needed [128, 152, 158–160]. Tumor necrosis factor antagonists (e.g., infliximab and etanercept) have failed to show clinical or serological improvement in patients with SS. Consequently, anti-TNFs are not recommended for treatment of SS, but may be indicated in selected SS patients where there is overlap with RA or other conditions where TNF inhibition therapy is indicated [128, 151]. Other biologic agents, such as anakinra (anti-IL-1), tocilizumab (anti-IL-6) and efalizumab (anti-CD11a), are not currently recommended for SS due to insufficient evidence of efficacy [128, 158].
